# Salivary levels of five microorganisms of root caries in nursing home elderly: a preliminary investigation

**DOI:** 10.1186/s12903-023-02953-9

**Published:** 2023-06-03

**Authors:** Lin Chen, Yuandong Qin, Yuhong Lin, Minquan Du, Yuhong Li, Mingwen Fan

**Affiliations:** 1 Stomatological Hospital, School of Stomatology, Southern Medical University, No. 366, South of Jiangnan Avenue, Guangzhou, China; 2 The State Key Laboratory Breeding Base of Basic Science of Stomatology (Hubei- MOST) & Key Laboratory of Oral Microbiology Ministry of Education, School & Hospital of Stomatology, Wuhan University, Wuhan, China; 3 Department of Endodontics, Yantai Stomatological Hospital Affiliated to Binzhou Medical College, Yantai, China; 4 First dental hospital , Jean Han University, Wuhan, China

**Keywords:** Root caries, Quantitative real-time polymerase chain reaction, Elderly, bacteria, Saliva

## Abstract

**Background:**

*Streptococcus*, *Bifidobacteria*, *Lactobacillus* and *Actinomyces* are acidogenic aciduria that may be associated with root caries (RC). The aim of the study was to analyze *Streptococcus mutans* (*S. mutans*), *Streptococcus sobrinus* (*S. sobrinus*), *Bifidobacterium* spp., *Lactobacillus* spp. and *Actinomyces naeslundii* (*A. naeslundii*) in the saliva of nursing home elderly, to assess the correlation between bacterial composition and RC for five putative catiogenic organisms.

**Methods:**

In this study, we collected 43 saliva samples and divided into two groups: the root caries group (RCG, n = 21) and the caries-free group (CFG, n = 22). Bacterial DNA was extracted from the saliva samples. The presence and abundance of the five microorganisms were detected by Quantitative real-time PCR (qPCR). Spearman correlation test was performed to evaluate the relationship between the numbers of root decayed filled surfaces (RDFS) and root caries index (RCI) and salivary levels of the bacteria.

**Results:**

The salivary levels of *S. mutans*, *S. sobrinus*, *Bifidobacterium* spp. and *Lactobacillus* spp. were significantly higher in RCG than in CFG (p < 0.05). RDFS and RCI (RDFS/RCI) were positively associated with salivary levels of *S. mutans*, *S. sobrinus* and *Bifidobacterium* spp. (r = 0.658/0.635, r = 0.465/0.420 and r = 0.407/0.406, respectively). No significant differences in presence and amounts of *A. naeslundii* was observed between the two groups (p > 0.05).

**Conclusion:**

*S. mutans*, *S. sobrinus* and *Bifidobacterium* spp. in saliva appear to be associated with RC in the elderly. Taken together, the findings indicate that specific salivary bacteria may be involved in the progression of RC.

## Background

The global elderly population is increasing. The China Seventh Population Census has shown that the number of older people aged 60 years and over has reached 264 million, accounting for 18.7% of the total population [1]. Multiple studies have reported a much higher prevalence of RC in older adults with dental problems than other adult populations [2]. RC is an important public oral health facing human beings due to the improvements of medical health care level and the extension of life expectancy, and the demand for maintaining oral health is increasing [3, 4] .

The pathogenesis of RC is affected by many factors, among which microorganisms play an important role [5]. There is no consensus on the microbial etiologic of RC in the elderly. Previous studies of the microbiota associated with RC have shown that bacteria associated with the disease include *Streptococcus*, *Actinomyces* and *Bifidobacteria* [6, 7]. Several reports have demonstrated that *S. mutans* is more prone to plaque formation on decaying surfaces compared to healthy root surfaces when used alone or in combination with *Lactobacillus* spp [8, 9]. Using molecular techniques, Preza et al. showed that the putative RC pathogens including *S. mutans*, *Lactobacillus* and *Actinomyces* and others [4]. Unfortunately, several studies have failed to link specific species to the etiologic of RC [10–12].However, comparing the results of different studies appears to be difficult due to differences in methods, samples, and analyses. Oral bacteria were identified using varieties of methods, including but not limited to culture, direct enzyme tests, enzyme-linked immunosorbent assays, denaturing gradient gel electrophoresis, DNA probes and 454-pyrosequencing. qRT-PCR has been used widely used for the detection of targeted microorganisms and the assessment of human oral bacterial colonization due to its advantages of reliability, rapidity, simplicity and economy [13–19].

Knowledge of the relationship between oral bacteriology and RC is limited, although several studies have qualitatively and quantitatively described the microbiology of RC in the elderly, almost all studies have focused on plaque rather than saliva [7, 20–22]. Furthermore, the temporal stability of the saliva microbiome and the easy availability of saliva were considered as the most appropriate probes to provide information about the entire oral microbial population [23, 24]. To our knowledge, few studies have used culture-independent assays to characterize distinct microbial population in the saliva of the elderly with RC. Therefore, the aim of this study was to identify five putative-cariogenic bacteria, namely *S. mutans, S. sobrinus*, *Bifidobacterium* spp., *Lactobacillus* spp. and *A. naeslundii* in the saliva of nursing home elderly with RC using qRT-PCR assay. We hypothesized that the five target microorganisms would be significantly more abundant in the saliva of the root caries group (RCG) than in the caries-free group (CFG). Secondly, we predicted that the amounts of these bacteria in saliva would correlate with the severity of RC.

## Methods

### Subject population

This study was approved by the Human Ethics Research Committee of the Hospital of Stomatology, Wuhan University (Approval No. 2011-0030). To have an 80% chance of detecting a difference of 0.2 as significant (at the two-side 5% level), a sample size of 15 subjects was calculated. Based on the assumption of a follow-up dropout of 20%,18 subjects per group were required. Finally, 43 subjects (19 women and 24 men) were recruited from two nursing homes in Wuhan (China) conducted in April,2018 in the study. Informed consent was obtained from all subjects. The inclusion criteria included a minimum age of 60 years, at least 20 existing natural teeth, willingness to participate in the research, no antibiotic therapy or professional cleaning within the past 3 months, not on immunosuppressant medications or steroids, no dry mouth symptoms, no diabetes or human immunodeficiency virus. The subjects were required to exhibit at least one root surface caries tooth to be included in RCG and no coronal caries or RC to be included in CFG.

### Clinical examination and questionnaires

All subjects were clinically examined with the aid of a mirror and a ball-ended WHO Community Periodontal Index probe by two calibrated dental epidemiologists in the dental clinic with the assistance of trained recorder. RC definition and diagnosis were based on the criteria of the World Health Organisation [25]. A lesion on an exposed root surface was classified as RC if it felt soft or leathery on probing. Clinical oral health status was measured using root decayed filled surfaces (RDFS) [25], root caries index (RCI) [26], the community periodontal index (CPI) and the number of remaining teeth (NRT). The kappa coefficients for the indices of RDFS and CPI were 0.80 and 0.77.

After clinical examination, questionnaires referring to the tooth brushing habits, sugar intake per week and smoking of each subject were recorded.

### Sampling and extraction of genomic DNA

Sampling was performed after oral examination. All subjects were asked to avoid eating or drinking for 1 h before oral sampling. Unstimulated saliva samples of at least 2 mL were collected from each subject. All saliva samples were packed in coolers with cold packs supplemented with ice and transported to the laboratory within 3 h, where they were frozen at − 80 °C until further analysis.

Total bacterial genomic DNA was extracted from each saliva sample using the modification of the Epicentre method (Epicentre, Madison, WI, USA) according to published protocol [27]. The final quantity and quality of DNA was evaluated using ND-1000 at OD260/OD280. A standard concentration of 10 ng/µL was prepared for each sample for all qRT-PCR assays.

### Bacterial strains and media

The five oral bacterial strains included *S. mutans* strain ATCC 700,610, *S. sobrinus* strain 6715, *Bifidobacterium dentium* (*B. dentium*) strain ATCC 27,534, *Lactobacillus* acidophilus (*L. acidophilus*) strain ATCC 4356 and *(A) naeslundii* strain ATCC 12,104. Strains of *(B) dentium* ATCC 27,534 (order No. ATCC 27,534) and *L. acidophilus* ATCC 4356 (order No. ATCC 4356) and genomic DNA of *S. mutans* ATCC 700610D (order No. ATCC 700610-5) and *A. naeslundii* ATCC 12104D (order No. ATCC 12104-5) were directly purchased from the American Type Culture Collection (ATCC, Manassas, VA, USA). *S. sobrinus* strain 6715 was purchased from China Center for Type Culture Collection (CCTCC, Wuhan, China). The selected stains were incubated under the anaerobic conditions at 37 °C using mediums of brain heart infusion (Becton Dickinson, NJ, USA), tryticase phytone glucose (Hopebio, Qingdao, China) and lactobacilli MRS (Becton Dickinson, NJ, USA). Genomic DNA preparations from each strain, except *S. mutans* and *A. naeslundii*, were obtained using the method above and then purified.

### qRT-PCR assays

Amplification and quantification were performed with an ABI 7500 system (Applied Biosystems, Foster City, CA, USA). The qRT-PCR mixture containing a total volume of 20 µL consisted of 10 µL of 2X SYBR Premix DimerEraser (Takara, Shiga, Japan), 0.4 µM of each forward and reverse primer, 0.4 µL of 50X ROX and 2.5 µL of the template DNA. Each sample and standard was tested in duplicate and the final analysis was based on the mean of the two reactions. The thermal cycling conditions for all qRT-PCR assays were as follows: 95 °C for 2 min, followed by 40 cycles at 95 °C for 5 s, 60 °C for 60 s for *S. mutans*, *S. sobrinus* and *Lactobacillus* spp. and *A. naeslundii*. The cycling conditions for *Bifidobacterium* spp. were as follows: 95 °C for 2 min, followed by 40 cycles at 95 °C for 5 s, 58 °C for 30 s and 72 °C for 60 s. The specific primers used for qRT-PCR are listed in Table 1.

**Fig. 1:**
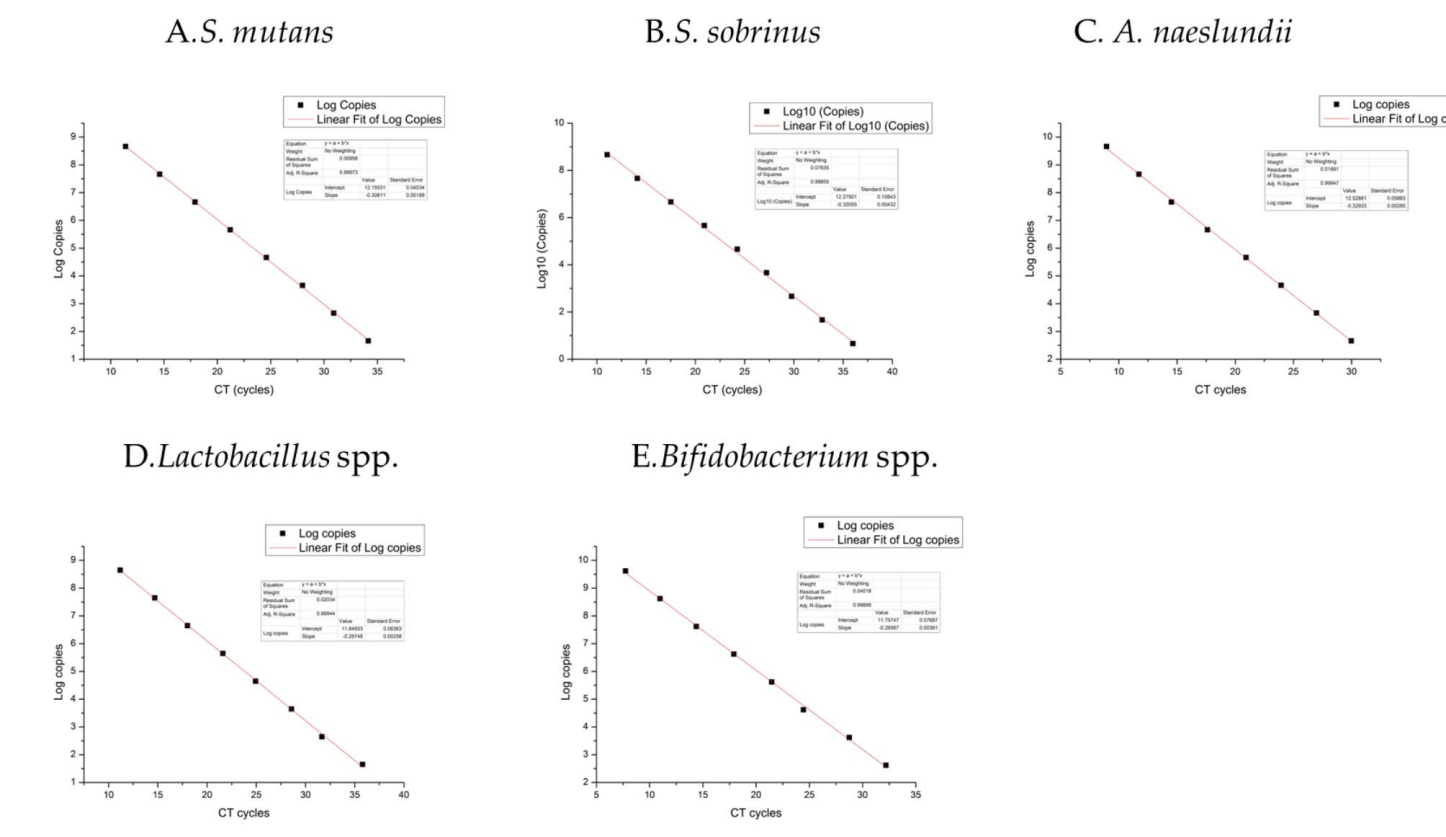
Standard curves of the known bacterial

The quantity of DNA was calculated from standard curves (Fig. 1) for each bacterial species, using DNA controls from standard bacterial cultures, which Were diluted from series of 10-fold. After the final cycle of qPCR, analysis of the cycle threshold (CT) and melting temperature (Tm) values were carried out in all the amplified samples. Samples were considered negative for bacterial species when their CT and Tm values were below the level of detection in the curves of DNA standards. All values were measured in duplicate and linearity was reproduced in a second run.

**Table 1:** Species-specific primers for real-time qPCR assay

Microorganisms	Primers (5’-3’)	Amplicon size	Reference
*S. mutans*	GCC TAC AGC TCA GAG ATG CTA TTCT GCC ATA CAC CAC TCA TGA ATT GA	114	Childers [28]
*S. sobrinus*	GGA CTT GCT CCA GTG TTA CTA ATG AG CCG CTA TCA GGC AGG TTA CC	88	Price [13]
*Bifidobacterium spp.*	GGG TGG TAA TGC CGG ATG CCA CCG TTA CAC CGG GAA	498	Mantzourani [29]
*Lactobacillus spp.*	TGG AAA CAG ATG CTA ATA CCG CGT CCA TTG TGG TAG ATT CCC T	223	Bizhang [20]
*A. naeslundii*	CTCCTACGGGAGGCAGCAG CACCCACAAACGAGGCAG	135	Dalwai [17]

### Statistical analysis

The qRT-PCR results were organised and analysed using the SPSS 19.0 statistical software package (SPSS Inc., Chicago, IL, USA). Descriptive statistics, including prevalence, mean, range, standard deviation, standard error and variance, were examined. Nonparametric Mann–Whitney U test was used to examine the significance of distribution among continuous variables. Fisher’s exact test was used to examine the significance of distribution among categorical variables. Spearman correlation test was used to examine the correlation coefficients between the salivary levels of bacteria and the indices of RDFS and RCI. A p value less than or equal to 0.05 was considered statistically significant.

## Results

### General results

Demographic and clinical characteristics of the study population was showed in Table 2. The mean NRT was significantly higher in CFG (25.8) than in RCG (23.7; p = 0.007). Age, gender, CPI scores, oral hygiene/day, sugar intake/week and smoking between the two groups were not statistically significant (p > 0.05).

**Table 2:** Demographic and clinical characteristics of the study population

	RCG (n = 21)	CFG (n = 22)	p values
General characteristics			
Mean age ± SEM ^a^ (years)	75.6 ± 1.5	73.4 ± 1.4	0.368
Gender female ^b^ (%)	42.9	45.5	1.000
Clinical characteristics			
Mean CPI ^a^	2.9 ± 0.1	2.5 ± 0.1	0.078
Mean NRT ^a^	23.7 ± 0.5	25.8 ± 0.5	0.007*
Survey characteristics			
Oral hygiene/day ^a^ (mean ± SEM)	1.8 ± 0.1	1.9 ± 0.1	0.404
Sugar intake/week ^a^ (mean ± SEM )	1.2 ± 0.6	0.9 ± 0.4	0.593
Smoking ^b^ (%)	9.5	9.1	1.000

SEM = standard error of the mean

NRT = number of remaining teeth

CPI = community periodontal index

^a^ Nonparametric Mann-Whitney U test

^b^ Fisher’s exact test.

(* p < 0.05, ** p < 0.01)

### Prevalence of the five bacteria in RCG and CFG

Table 3 shows the prevalence of the five targeted bacteria. Among the five bacteria examined, *S. sobrinus* had significantly higher prevalence in RCG (47.6%) than in CFG (18.2%; p = 0.039). Whilst the positive prevalence of *S. mutans* and *Bifidobacterium* spp. in RCG was higher than that in CFG, differences between the two microorganisms were not statistically significant (p = 0.079 and p = 0.052, respectively). *Lactobacillus* spp. and *A. naeslundii* were positive in all of the subjects.

**Table 3:** Prevalence of the five targeted microorganisms in RCG and CFG saliva

	RCG (n = 21)	CFG (n = 22)	p values
***S. mutans*** ***S. sobrinus*** ***Bifidobacterium spp.*** ***Lactobacillus spp.*** ***A. naeslundii***	100% 47.6% 85.7% 100% 100%	86.5% 18.2% 59.1% 100% 100%	0.079 0.039* 0.052 1.000 1.000

Fisher’s exact test. (* p < 0.05, ** p < 0.01)

**Fig. 2:**
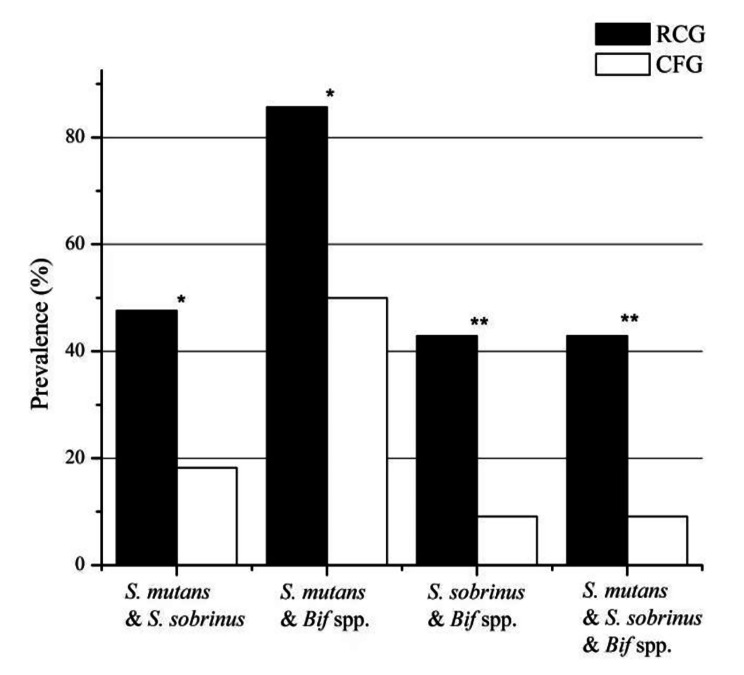
Co-prevalence of bacterial taxons from saliva in RCG and in CFG. The co-occurrences of *S. mutans* & *S. sobrinus*, *S. mutans* & *Bifidobacterium* spp., *S. sobrinus* & *Bifidobacterium* spp. & *S. mutans*, *S. sobrinus* & *Bifidobacterium* spp. were found to be significantly more frequent in RCG than in CFG (p < 0.05). (* p < 0.05, ** p < 0.01)

We further analysed the co-prevalence of bacterial taxons from saliva in RCG and in CFG (Fig. 2). The co-occurrences of *S. mutans* & *S. sobrinus*, *S. mutans* & *Bifidobacterium* spp., *S. sobrinus* & *Bifidobacterium* spp. & *S. mutans*, *S. sobrinus* & *Bifidobacterium* spp. were found to be significantly more frequent in RCG than in CFG (p < 0.05).

### Salivary levels of the five targeted microorganisms in RCG and CFG

The salivary levels of the five targeted microorganisms between the two groups were evaluated (Table 4). The salivary levels of all five targeted bacteria in RCG were higher than those in CFG. *S. mutans*, *S. sobrinus*, *Bifidobacterium* spp. and *Lactobacillus* spp. were significantly higher in RCG than in CFG (p < 0.05). No significant differences in *A. naeslundii* were observed between the two groups (p > 0.05).

**Table 4:** Comparison of the mean DNA levels log_10_ (cells/ml + 1) of targeted microorganisms between the RC and CFG

Microorganisms	Mean ± SEM		
	**RCG (n = 21)**	**CFG (n = 22)**	**p values**
***S. mutans*** ***S. sobrinus*** ***Bifidobacterium spp.*** ***Lactobacillus spp.*** ***A. naeslundii***	6.0 ± 0.2 2.5 ± 0.6 5.7 ± 0.5 8.8 ± 0.1 8.6 ± 0.1	4.1 ± 0.4 0.6 ± 0.3 3.7 ± 0.7 8.6 ± 0.2 8.6 ± 0.1	0.000** 0.018* 0.008** 0.044* 1.000

Nonparametric Mann-Whitney U test.

(* p < 0.05, ** p < 0.01)

The relative ratio of each species within the total five species from RCG and CFG was shown in Table 5. *A. naeslundii* showed higher ratio in CFG than in RCG. By contrast, the four other bacteria showed greater ratio in RCG than in CFG.

**Table 5:** Frequencies of each of the five species in RCG and CFG saliva

Microorganisms	Mean ± SEM		
	**RCG (n = 21)**	**CFG (n = 22)**	**p values**
***S. mutans*** ***S. sobrinus*** ***Bifidobacterium spp.*** ***Lactobacillus spp.*** ***A. naeslundii***	0.37 ± 0.16 0.26 ± 0.01 1.08 ± 0.56 61.36 ± 4.87 36.93 ± 4.91	0.02 ± 0.01 0.00 ± 0.00 0.42 ± 0.21 51.53 ± 5.00 48.02 ± 4.94	0.000** 0.021* 0.019** 0.198 0.145

Nonparametric Mann-Whitney U test

(* p < 0.05, ** p < 0.01)

### Correlation between the five bacteria and RDFS index and RCI in both groups

The correlation between the RDFS index and RCI and salivary levels of bacteria were calculated. The correlation coefficients (r) between RDFS and *S. mutans*, *S. sobrinus*, *Bifidobacterium* spp., *Lactobacillus* spp. and *A. naeslundii* were 0.658, 0.465, 0.407, 0.271 and − 0.065, respectively. As well, r values between RCI and the corresponding microorganisms were 0.635, 0.420, 0.406, 0.252 and − 0.044, respectively (Table 6).

**Table 6:** Correlations between the RDFS index and RCI and salivary levels of bacteria

Microorganisms	r	
	**RDFS**	**RCI**
***S. mutans*** ***S. sobrinus*** ***Bifidobacterium spp.*** ***Lactobacillius spp.*** ***A. naeslundii***	0.658** 0.465** 0.407** 0.271 0.027	0.635** 0.420** 0.406** 0.252 0.011

r: correlation coefficients were calculated using Spearman’s test

(* p < 0.05, ** p < 0.01)

## Discussion

RC is a significant oral health issue, especially in the elderly. Detailed information on the composition of oral microbiota in relation to dental caries may aid in assessing the individual’s risk and better understanding the etiology. Here, we used qRT-PCR to analyze the bacterial composition of five putative-cariogenic microorganisms in the saliva of nursing home elderly subjects with RC for the first time.

For a long time, RC was thought to be induced specifically by *Actinomyces* [6, 30]. *A. naeslundii*, one of the most common species of *Actinomyces*, has been demonstrated to be related to RC [10, 31]. In contrast, other studies have showed that *A. naeslundii* might play beneficial roles as well [32, 33]. Other study indicate that some bacteria, frequently related to oral health, have been also involved with dental caries, then being considered as alternative pathogens such as *A. naeslundii* [33]. Interesting, in the present study, no statistical difference was observed among the two groups for *A. naeslundii*, which is consistent with previous literature reports [19]. *A naselundii* has been considered as probiotics because some of their metabolic activities may modulate dynamic caries processes in a different way, such as the use of lactate as a carbon source [34]. Another study showed that *A. naeslundii* may have inhibited *S. mutans* growth [35]. Consequently, the molecular mechanisms through which these microorganisms participate in caries initiation remain unclear and further studies are needed to identify the ecological shifts leading to cariogenic biofilms [36].

Mutans streptococci (MS, *S. mutans* and *S. sobrinus*) were the investigated carious bacteria in the past [8, 9, 14–16, 18]. In the present study, both *S. mutans* and *S. sobrinus* were detected. Our results showed that the prevalence rates of *S. mutans* and *S. sobrinus* were higher in RCG than in CFG, but a statistically significant difference was found only for *S. sobrinus* (Table 3). Meanwhile, we observed that the co-prevalence of *S. sobrinus* and *S. mutan* was 48% in our study. Similar results have been described in the Oda et al.’ study, they report that both *S.mutans* and *S.sobrinus* were found positive in 58% individuals [37]. However, the results of different studies are still controversial. In accordance with N.M. Nurelhuda et al.’ study, *S. sobrinus* was never present alone and was always detected alongside with *S. mutans* [15]. While Franco et al. reported zero prevalence of both the species residing together in study individuals [38]. In our qRT-PCR analyses, significantly higher amounts of *S. mutans* and *S. sobrinus* were observed in RCG than in CFG (Table 4), which is in accordance with results of previous studies on coronal caries [14, 39] and RC [20]. In addition, we found that the salivary levels of *S. mutans* and *S. sobrinus* were positively correlated with the indices of RDFS/RCI (Table 6), implying that abundance of *S. mutans* and *S. sobrinus* increased with severity of RC. These findings suggest that *S. mutans* and *S. sobrinus* may be involved in some progression of RC and hence considered as meaningful bio-markers for assessing the risk of RC.

*Bifidobacteria* are acidogenic and aciduric microorganisms that have recently been verified to be related to coronal caries in children and adults [19, 29], as well as clinical severity of RC [7]. As shown in Fig. 1, the co-prevalence of *S. mutans* & *Bifidobacterium* spp., *S. sobrinus* & *Bifidobacterium* spp. and *S. mutans* & *S. sobrinus* & *Bifidobacterium* spp. were manifested significantly higher in RCG than in CFG. Therefore, co-occurrence with multi-bacteria may be valuable in risk assessment of dental caries, which is consistent with the findings of Tanner et al [40]. Using culture-based methods, Mantzourani et al. and Kaur et al. found that *Bifidobacteria* were positively related to the RC in the plaque [7] and coronal caries in the saliva [39], respectively. Our observation that the amounts of *Bifidobacterium* spp. were significantly higher in RCG than in CFG validates this finding. We also found that the severity of RC measured by RDFS/RCI correlated positively with the salivary levels of *Bifidobacterium* spp. Taken together, these findings confirm our hypothesis and suggest that *Bifidobacterium* spp. may contribute to the etiologic of RC. Actually, Species in *Scardovia*, a genus in the *Bifidobacterium* family, have been identified in cavitated dentin lesions in addition to *Streptococcus* and *Lactobacillus* [41]. Santos et al. found that associations of *B*. *animalis* and *B. longum* with *streptococci* promoted EPS production and caries lesion progression [42]. These observations give importtant insights into the influences between *Bifidobacteria* and *streptococci*, which should be addressed in the future.

Among the five selected microorganisms analysed in this study, the highest bacteria load was that of *Lactobacillus* spp. (Table 4). *Lactobacilli* have been extensively reported to be involved in caries progression [43–45]. Our quantitative results demonstrated that the salivary level of *Lactobacillus* spp. was significantly higher in RCG than in CFG (Table 5), which does agree with previous studies [20, 29]. However, the salivary level of *Lactobacillus* spp. did not correlate positively with the indices of RFDS and RCI (Table 6), indicating that the abundance of *lactobacilli* did not increase with the growing severity of RC. This is consistent with previous studies [18, 19]. Contrastly, Lapirattanakul et al. found that the detection of oral *lactobacilli* together with *S. mutans* was related to highest dental caries severity and *Lactobacillus fermentum* was the most prevalent, and its presence was related to high scores of caries [46]. Nevertheless, it should be noted that more well-designed studies aimed at specific species of *lactobacilli* are necessary to further test the relationship between the bacteria and RC.

qRT-PCR analysis provided a sensitive, rapid, reliable and simple method for quantification of bacteria. Although specific primers were eligible for identification of the target microorganisms, our results may still be confounded by primer bias. For example, in the case of *Bifidobacterium* spp., the primers not only cover the targeted genera, but also recognize other oral *Bifidobacteria* such as *Scardovia*, *Parascardovia* and *Gemella*. Another limitation of this study is that only five microorganisms were selected, which may not be sufficient to uncover the relation between all the microorganisms and RC. Although our data are valid and substantial enough to meet the aim of our study, further studies involving more taxons are needed and recommended.

## Conclusions

In summary, using qRT-PCR assay, we first demonstrated bacterial composition of five bacteria in the saliva of elderly with RC. The first study hypothesis that the selected putative-cariogenic microorganisms would be found to be significantly more abundant in RCG than CFG was confirmed for *S. mutans*, *S. sobrinus*, *Bifidobacterium* spp. and *Lactobacillus* spp. The second study hypothesis that the amounts of five targeted bacteria would be associated with the severity of RC was confirmed only for *S. mutans*, *S. sobrinus* and *Bifidobacterium* spp. Based on our results, *S. mutans*, *S. sobrinus* and *Bifidobacterium* spp. in saliva appear to be associated with RC in the elderly and may act as bio-markers for the risk assessment of RC.

## Data Availability

The datasets used and analyzed during the current study are available from the corresponding author on reasonable request.

## List of abbreviations

RC: Root caries
RCI: Root caries index
NRT: Number of remaining teeth
RDFS: Root decayed filled surfaces
RCG: Root caries group
CFG: Caries-free group
CPI: Community periodontal index
qPCR: Quantitative real-time PCR
*S. mutans*: *Streptococcus mutans*
*S. sobrinus*: *Streptococcus sobrinus*
*A. naeslundii*: *Actinomyces* *naeslundii*
*B. dentium*: *Bifidobacterium dentium*
*L. acidophilus*: *Lactobacillus acidophilus*
